# Nuclear Shape Changes Are Induced by Knockdown of the SWI/SNF ATPase BRG1 and Are Independent of Cytoskeletal Connections

**DOI:** 10.1371/journal.pone.0055628

**Published:** 2013-02-06

**Authors:** Karen M. Imbalzano, Nathalie Cohet, Qiong Wu, Jean M. Underwood, Anthony N. Imbalzano, Jeffrey A. Nickerson

**Affiliations:** Department of Cell and Developmental Biology, University of Massachusetts Medical School, Worcester, Massachusetts, United States of America; George Mason University, United States of America

## Abstract

Changes in nuclear morphology occur during normal development and have been observed during the progression of several diseases. The shape of a nucleus is governed by the balance of forces exerted by nuclear-cytoskeletal contacts and internal forces created by the structure of the chromatin and nuclear envelope. However, factors that regulate the balance of these forces and determine nuclear shape are poorly understood. The SWI/SNF chromatin remodeling enzyme ATPase, BRG1, has been shown to contribute to the regulation of overall cell size and shape. Here we document that immortalized mammary epithelial cells show BRG1-dependent nuclear shape changes. Specifically, knockdown of BRG1 induced grooves in the nuclear periphery that could be documented by cytological and ultrastructural methods. To test the hypothesis that the observed changes in nuclear morphology resulted from altered tension exerted by the cytoskeleton, we disrupted the major cytoskeletal networks and quantified the frequency of BRG1-dependent changes in nuclear morphology. The results demonstrated that disruption of cytoskeletal networks did not change the frequency of BRG1-induced nuclear shape changes. These findings suggest that BRG1 mediates control of nuclear shape by internal nuclear mechanisms that likely control chromatin dynamics.

## Introduction

The SWI/SNF complexes comprise a family of ATP-dependent chromatin remodeling enzymes that utilize the energy released from ATP hydrolysis to break or destabilize histone-DNA contacts on the nucleosome [1],[2]. Numerous structural alterations are possible following SWI/SNF mediated remodeling [3],[4]. The functional consequence of these chromatin structure changes is increased accessibility of regulatory and enzymatic proteins that modulate chromatin assembly, DNA replication, repair and recombination, and transcription [3]. SWI/SNF complexes are evolutionarily conserved in eukaryotes and contain either BRM (Brahma) or BRG1 (Brahma-related gene 1) as an essential ATPase subunit [5]–[7]. SWI/SNF complexes include other proteins known as BRG1 and BRM-associated factors (BAFs) that can modulate the activity of BRM or BRG1 in a gene-specific fashion [7].

To address the function of the SWI/SNF ATPases in normal mammary epithelial cells, we generated inducible knockdowns of either BRG1 or BRM in the non-tumorigenic mammary epithelial cell MCF-10A [8]. The depletion of either ATPase subunit decreased the rate of cell proliferation without inducing either apoptosis or complete growth arrest in monolayer culture or in three-dimensional reconstituted basement membrane (rBM) cultures. The length of the cell cycle increased after depletion of either SWI/SNF ATPase, indicating a role for BRG1 and BRM as positive regulators of proliferation at all stages of the cell cycle. These results were unexpected since mice heterozygous for the BRG1 gene have an increased risk of mammary carcinoma [9]–[11].

We report here that the depletion of BRG1, but not of BRM in immortalized but non-transformed mammary epithelial cells induced nuclear shape changes, including lobulation and the development of folds in the nuclear surface. Paradoxically, these changes in nuclear shape were similar to changes in nuclear structure often observed in tumors [12], including breast tumors [13],[14]. Similar changes have also been observed in the laminopathies, diseases caused by the mutation of Lamin A or its interacting structural proteins at the nuclear periphery [15],[16]. Exploration of these diseases has demonstrated the critical importance of the architecture of the nuclear periphery, the place where the nuclear lamina, nuclear envelope, nuclear lamina-associated chromatin, and the cytoskeleton all intersect. Disruption of these structures may have profound effects on cellular mechanics and on gene expression. Multiple proteins of the nuclear lamina and nuclear envelope bind to chromatin (reviewed in [17]). The genes packaged in the peripheral heterochromatin attached to the nuclear lamina are predominantly silenced; release of chromatin from the lamina toward the nuclear interior may facilitate the activation of such genes [17].

Nuclear shape may be determined in part by connections between the peripheral structures of the nucleus and the cytoskeleton. Forces generated by the cytoskeleton would keep the nucleus under tension at each connection, and the balance of tensions would influence nuclear shape. The continuity between cell surface integrins, the cytoskeleton, and the nucleus was directly demonstrated in living endothelial cells by micromanipulation [18]. The nucleus in differentiated cells is under tension mediated by a pre-stressed cytoskeleton [19],[20]. Early studies showed that the intermediate filaments were connected to the nucleus in epithelial cells where they are heteropolymers of at least two cytokeratins [21]–[24], in fibroblasts where they are polymers of vimentin [22],[25], and in a subset of neurons that have intermediate filaments containing peripherin [26]. Electron microscopy revealed filaments of 5 to 6 nm in diameter connecting 10 nm cytokeratin filaments to the nuclear lamina [27]. Nuclear-anchored cytokeratin filaments attach to desmosomes and to other structures at the plasma membrane, linking the nucleus through the intermediate filaments to the cell surface [22],[24].

More recent work characterizing the molecular details of these connections has identified the LINC (Linker of Nucleoskeleton and Cytoskeleton) complex [28],[29]. In this complex, the Nesprin proteins located at the nuclear envelope bind to cytoskeletal filaments, cross the outer nuclear membrane, and bind the SUN1 and SUN2 proteins in the lumen of the nuclear envelope. The SUN proteins, which form a heterodimer, bind to lamins in the nuclear lamina, to other proteins associated with the nuclear lamina and inner nuclear membrane, and to the chromatin that is located at the inner surface of the nuclear lamina. Different members of the Nesprin protein family bind to actin filaments of the cytoskeleton (Nesprin 1 and 2) [30],[31], to plectin cross-bridges with intermediate filaments (Nesprin 3) [32],[33], or to the microtubule associated motor protein kinesin (Nesprin 4) [34]. It may be the plectin cross-bridges between intermediate filaments and Nesprin 3 that were earlier observed as 5 to 6 nm short filaments by electron microscopy [27]. The binding of Nesprins to SUN proteins is necessary for effective force transmission between the nucleus and cytoskeleton [35].

We initially hypothesized that the changes in nuclear shape that were caused by reduction in BRG1 levels might arise from an altered balance of tensions at connections between the cytoskeleton and the nuclear lamina, changes downstream of altered expression of cytoskeleton or LINC complex proteins. In order to directly test this hypothesis, we disrupted microtubules, actin filaments, or cytokeratin filaments in MCF-10A cells engineered with a doxycycline inducible knockdown of BRG1. Even after each cytoskeletal disruption, nuclear profiles were significantly altered by the reduction in BRG1 protein levels. We conclude that the BRG1 related changes in the architecture of the nuclear lamina, nuclear envelope, and peripheral heterochromatin are not caused by altered tension exerted on the nucleus by the cytoskeleton, but rather from BRG1-related changes in internal nuclear forces.

## Materials And Methods

### Cell Culture

MCF-10A cells were obtained from the Karmanos Cancer Center (Detroit, Michigan). MCF-10A and MCF-10A derived cells were cultured in phenol red free DMEM/F12 media supplemented with cholera toxin, epidermal growth factor, insulin, and hydrocortisone as described [8],[36]. MDA-MB-231 [37],[38] and MDA-MB-231 derived cells were cultured in DMEM containing 10% fetal bovine serum (Invitrogen, Carlsbad, CA). Knockdown was induced by addition of 10 ng/µl doxycycline (Sigma-Aldrich, St. Louis, MO).

### Vectors, Lentivirus Production, And Generation Of Cell Lines

Vector generation, lentivirus production, and transduction of cells were described [8]. Briefly, MCF-10A or MDA-MB-231 cells were transduced with a lentivirus expressing the tTR-KRAB repressor and ds-Red fluorescent protein and fluorescence activated cell sorting (FACS) sorted to capture the ds-Red expressing population of cells. These cells were subsequently transduced with a doxycycline-inducible vector (PLVTHM; Addgene, Cambridge, MA) expressing a non-specific shRNA or a shRNA against either BRG1 or BRM [8] and green fluorescent protein (GFP). FACS sorting captured the ds-Red and GFP expressing cells, which were then verified for BRG1 or BRM knockdown by western blot. Virus was produced using Lipofectamine 2000 reagent (Invitrogen, Carlsbad, CA) in 293T cells.

### Sirna Analysis In Mcf-10a Cells

MCF-10A cells were independently transfected with one of three siRNAs to BRG1 (Invitrogen #1-216547A01, #2-216547A05, #3-21547A03) using Lipofectamine 2000 following the manufacturer’s instructions. A control scrambled siRNA was used (Thermo Scientific Dharmacon - 21 base pair sequence AACAGUCGCGUUUGCGACUGG). Transfection efficiency was monitored with a Santa Cruz control siRNA (fluorescein conjugate)-A (sc-36869). The transfections were done in duplicate. Media was changed 6 hours after transfection and cells were harvested after 72 hours for Western blots and fixed for immunofluorescence.

### Western Blot Analysis

Whole cell extracts from MCF-10A derived cells were prepared as described [8] from cells grown in the presence or absence of doxycycline for 3 days. The supernatant was separated by SDS-PAGE, and then transferred to nitrocellulose. MDA-MB-231 derived cells were lysed in 1× PBS containing 1% NP-40, 10 mM DTT, protease and phosphatase inhibitors cocktail (Roche, Indianapolis, IN) for 30 min at 4°C. Whole cell lysate was separated on a 4–20% SDS-PAGE (BioRad, Hercules, CA), and transferred to a PVDF membrane. Primary antibodies used were BRG1 (1∶1000, de la Serna et al, 2000), HP1γ (1∶500, Cell Signaling, Danvers, MA, 2619), PI3Kinase p85, H-SH2 domain (1∶1000, Millipore, Billerica, MA 06-496), GAPDH (1∶50000, Sigma-Aldrich, St. Louis, MO, G9295), and BRM (1∶1000; Abcam, Cambridge, MA, ab15597). GelQuant.NET software provided by biochemlabsolutions.com was used to quantify Western blots.

### Papanicolaou Staining And Immunofluorescence

Papanicolaou stain was used to analyze the cell morphology of the MCF-10A and MCF-10A derived cell lines according to the classical method previously described [39]. Cells were grown on cover slips in 6-well dishes, and after induction with doxycycline for 6 days, were washed once with PBS, and then fixed by a direct immersion in 95% ethanol. Cells were stained successively with Haematoxylin for 60 seconds, Orange G for 30 seconds, and Eosin Azure for 2 minutes. Cells were rinsed with ethanol between each staining and given a final rinse with Xylene before mounting. Analyses were performed with a Zeiss Axoplan optical microscope.

### Cytoskeletal Filament Disruption

Cells were treated with Latrunculin B (EDM Chemicals, Newark, NJ - 428020) at 0.2 µg/ml for 30 minutes, with Colcemid (CalbioChem, San Diego, CA –234109) at 0.05 µg/ml for 3 hours, or with sodium orthovanadate (Sigma-Aldrich, Saint Louis, MO – S6508) at 0.2 µg/ml for 30 minutes. Treated cells were then permeabilized for 10 minutes, ensuring the loss of constitutive dsRed and GFP, and then fixed following previously published methods [40].

### Immunofluorescence

For immunofluorescence studies, the primary antibodies used were against α-Tubulin (1∶400, Sigma-Aldrich, St. Louis, MO, T-5168), Cytokeratin 18 (1∶50, Thermo-Scientific, Waltham, MA, MS-142), swine Lamin A/C clone 638 (1∶100, Gene Tex, Irvine CA, GTX72069), HP1γ(1∶1000, Millipore, Billerica, MA, MAB3450), Lamin B (1∶500, Millipore, Billerica, MA, Clone 101-B7, Cytokeratin 8 (1∶50, Abcam, ab2531), Emerin (1∶50, Santa Cruz Biotechnology,sc-15378), NuMA (1∶40, Millipore, Billerica, MA, 204-41), phospho-Histone H3 (Ser10),(1∶1000, Millipore, Billerica, MA, 05-598), Histone H1,(1∶100, Millipore, Billerica, MA, P10412), Histone H3 (1∶100, Abcam, Cambridge, UK, ab8580), Nesprin 1(1∶50, Abcam, Cambridge, MA ab5250), Nurim (1∶50, Santa Cruz Biotechnology, Santa Cruz, CA, sc-133262) H3K9 trimethylated (1∶100, Millipore, Billerica, MA, 17625), SRm160 splicing factor clone B1C8 (1∶100, Millipore, Billerica, MA), HMGN2 (1∶100, Millipore, Billerica, MA, 07252), Paxillin (1∶100, BD Biosciences, San Jose, CA, P13520/L16), Vimentin (1∶200, Sigma-Aldrich, St. Louis, MO,V-5255), and PML (1∶500, Millipore, Billerica, MA, AB1370). Appropriate secondary antibodies (GAR-IgG-488, GAR-IgG-568, GAM-IgG-488, GAM-IgG-568) were from Molecular Probes (Eugene, OR) and used at 1∶2000. Nuclei were counter stained with DAPI (4′, 6-diamidino-2-phenylindole) (1∶4000 to 1∶6000, Invitrogen, Carlsbad, CA, D3571) and DRAQ 5 (1∶2000 to 1∶4000, BioStatus Limited, Leicestershire, UK, DR50050). Actin filaments were detected with Phalloidin-TRITC (1∶1000, Sigma-Aldrich, St. Louis, MO, P-1951). Cells were scored and micrographs were taken using a Zeiss Axoplan optical microscope or a Leica laser scanning confocal microscope (SP1 or SP5-II AOBS).

### Quantification And Statistical Analysis

A minimum of 200 and a maximum of 1000 nuclei were scored for each condition. Means plus or minus standard deviations are reported. Analysis of variance was determined with a Student’s t-test.

### Electron Microscopy

Electron microscopy was performed as described [41]. MCF-10A cells were grown on Thermanox coverslips at 37°C as described above. Cells were then fixed in 2.5% glutaraldehyde in either 0.1 M sodium cacodylate or Sorensen’s phosphate buffer, pH 7.4 for 1 hour at 4°C, followed by an additional 1–2 hours at room temperature. Samples were post-fixed in 1% osmium in the same buffer as the fix at 4°C for 1 hour, washed, dehydrated in graded ethanol’s, and embedded in Epon with propyleneoxide as the transitional solvent. Some blocks were re-embedded at 90 degrees to the growth surface so that transverse sections could be prepared and imaged. Images were taken with a Philips CM10 transmission electron microscope with an image intensified CCD camera.

## Results

### Identification Of Brg1-Dependent Nuclear Structure

**Figure 1:**
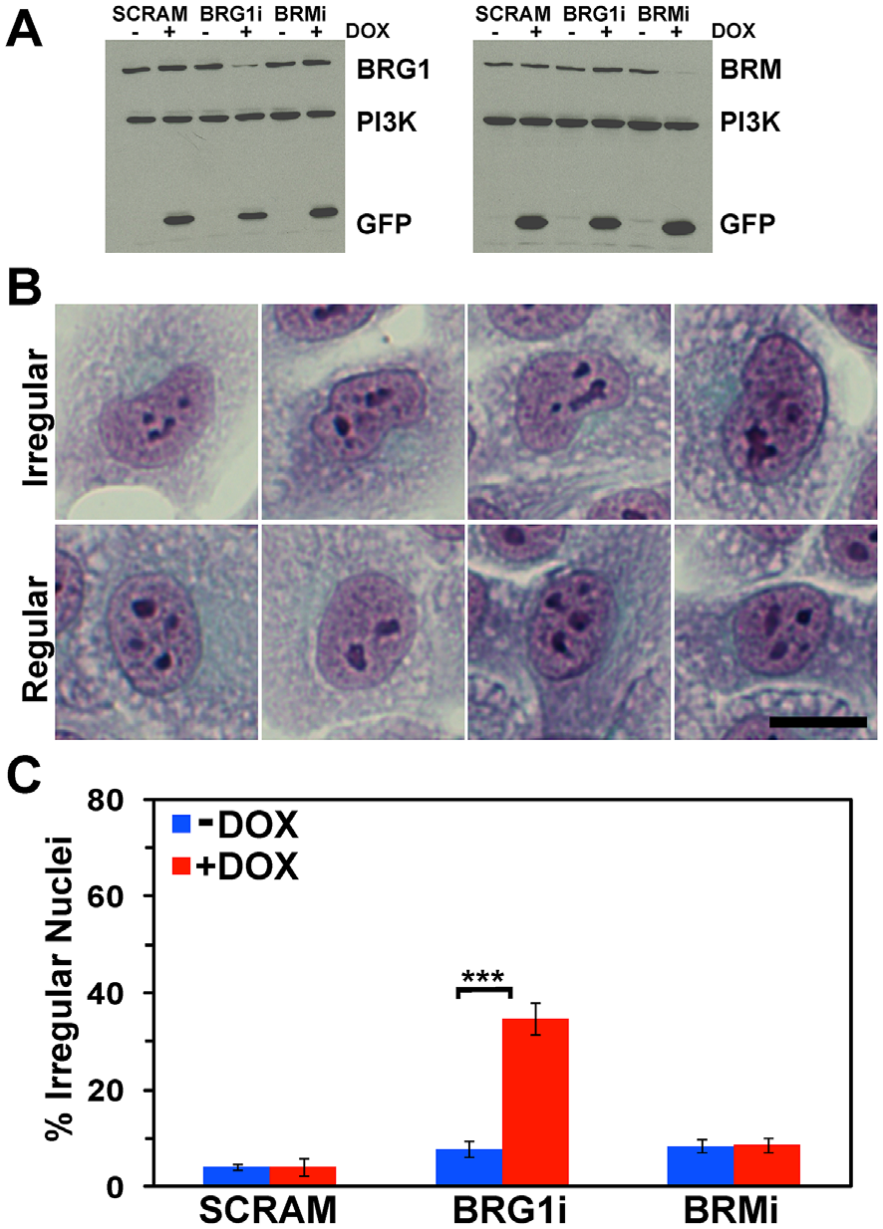
BRG1 knockdown in MCF-10A cells increases the frequency of irregularly shaped nuclei. (A) Western blot measuring the extent of BRG1 and BRM knockdown. PI3 Kinase was measured as a loading control. Induction of BRG1 or BRM knockdown correlates with Doxycycline (DOX) dependent GFP expression. (B) Representative images of irregular and regular shaped nuclei in PAP stained cells. Size bar 50 µm. (C) Quantification of irregularly shaped nuclei in MCF-10A cells upon BRG1 or BRM knockdown. SCRAM represents the control cells expressing a scrambled sequence shRNA. Data represent the means of 6 counts totaling a minimum of 200 (SCRAM +/− DOX), 290 (BRG1i +/− DOX), or 325 (BRMi +/− DOX) cells scored per cell line +/− standard deviation. *** represents p<0.005 as determined by Student’s t-test.

MCF-10A cells inducibly expressing a non-specific shRNA or shRNA against BRG1 or BRM (BRG1i and BRMi) have been previously described [8]. Western analysis for BRG1 and BRM expression confirmed the alteration in expression of these proteins (Figure 1A). Papanicolaou (PAP) staining is a multi-chromatic cell staining technique used in clinical cytology [39],[42],[43]. The many different colors and combinations of colors highlight many different features of cell architecture from chromatin condensation to cytokeratin expression. PAP staining of MCF-10A cells with inducible reduction of BRG1 levels identified many cells with altered nuclear shape, though there did not appear to be any significant changes in overall cell shape or size, or nuclear size. Irregularly shaped nuclei had clear and quantifiable features including curves and bulges that deviated from an oval profile and folds or invaginations in the structures of the nuclear surface. Some were lobulated sufficiently to have a kidney shape (Figure 1B). In contrast, oval shaped nuclei were consistently observed in the uninduced cells with normal levels of BRG1 (Figure 1B) as well as in both uninduced and induced cells expressing a scrambled sequence shRNA or the shRNA against BRM (data not shown). About one-third of the nuclei in the BRG1 knockdown cells were irregularly shaped, while ∼5% of the control cells showed an irregular nuclear appearance (Figure 1C). Less than 10% of the nuclei in the scramble control and BRM knockdown cells were irregular in shape, and the percentage of cells with irregular nuclei did not change in the presence of doxycycline. The results indicate that BRG1, but not BRM, plays a role in maintaining the normal nuclear shape.

### Brg1-Dependent Nuclear Grooves

**Figure 2:**
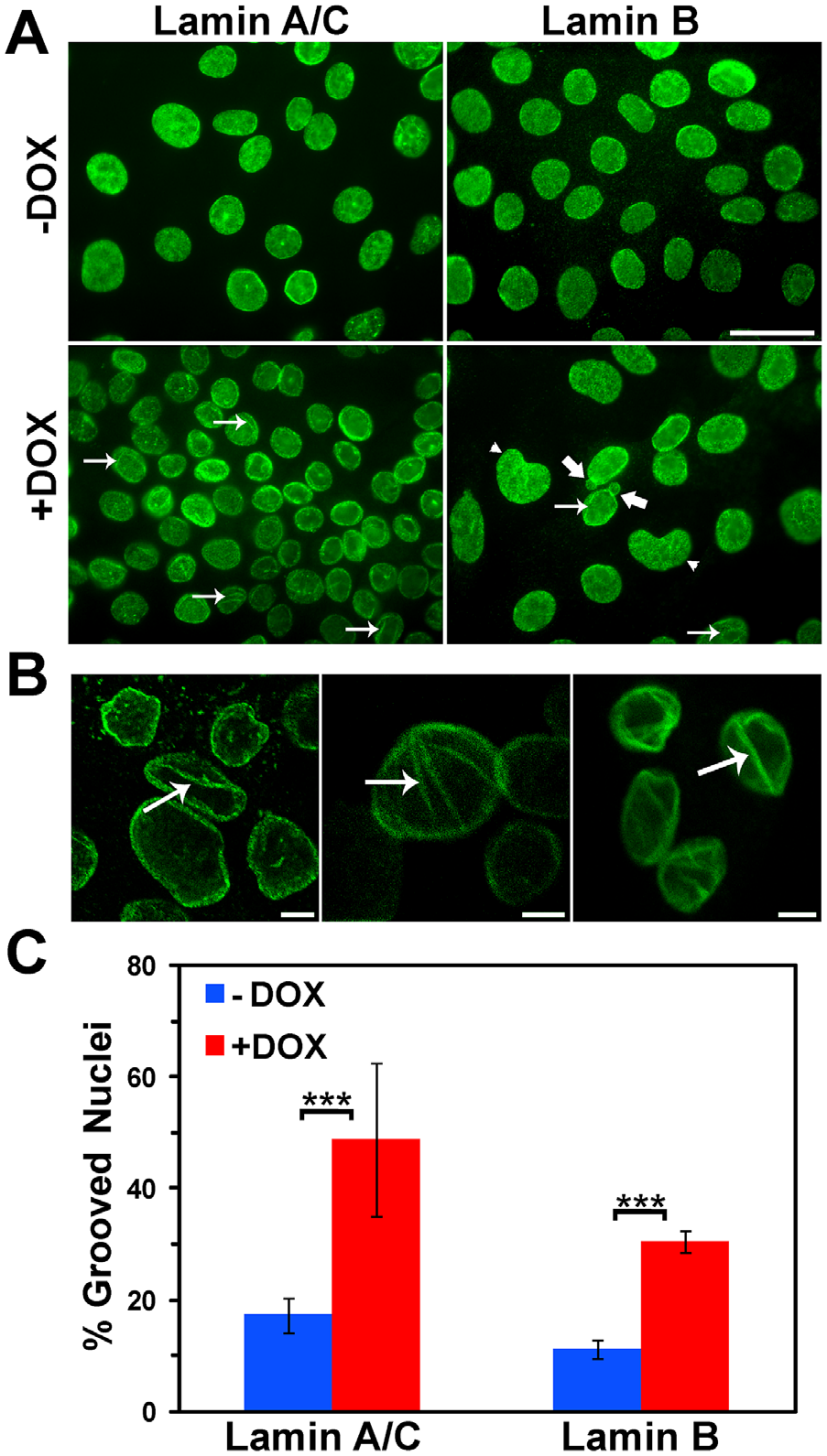
Increased incidence of nuclear grooves occurs in BRG1 knockdown MCF-10A cells. (A) MFC-10A cells in the presence or absence of BRG1 knockdown are shown labeled with either Lamin A/C or Lamin B. Arrows indicate grooves. Thick arrows indicate nuclear blebs. Arrowheads indicate kidney shaped nuclei. Size bar 10 µm. (B) Representative individual confocal sections of nuclei with grooves labeled with either Lamin A/C (center panel) or Lamin B (right and left panels). Arrows indicate grooves. Size bars 5 µm. (C) Quantification of grooved nuclei in MCF-10A cells upon BRG1 knockdown as identified by Lamin A/C or Lamin B staining. Data represent ten counts of at least 100 nuclei scored for grooves with Lamin A/C and three counts of at least 100 nuclei scored for grooves with Lamin B. Both are reported as means +/− standard deviation. *** represents p<0.005 as determined by Student’s t-test.

Since the irregular features of nuclei in BRG1 knockdown cells suggested structural alterations in the nuclear periphery, we examined the morphology of the nuclear lamina. The nuclear lamina of higher eukaryotes is a tightly woven meshwork of filaments connected to the inner nuclear membrane. The lamina is constructed from Lamin B proteins joined, in differentiated cells, by Lamins A and C, which are alternatively spliced from the same gene [44],[45]. Confocal imaging of both Lamin A/C and Lamin B revealed intensely staining nuclear grooves (arrows) in the BRG1 knockdown cells at an elevated frequency compared to the control cells cultured in the absence of doxycycline (Figure 2A) without any apparent difference in nuclear size. In addition to the presence of grooves, the Lamin A/C and Lamin B staining also identified the presence of kidney-shaped nuclei, (arrow head) as well as nuclei with blebs (thick arrow). Enlarged images are shown in Figure 2B. Quantification of the Lamin B stained cells indicated that approximately 30% of the nuclei in the BRG1 knockdown cells had grooves as compared to approximately 10% of the nuclei in the control cells (Figure 2C). Quantification of Lamin A/C stained cells indicated that nearly half of the nuclei showed evidence of grooves upon BRG1 knockdown, however, the background also rose, ranging between 15–20%. Though the frequency of grooved nuclei in BRG1 knockdown cells varied depending on whether the cells were stained for Lamin B or Lamin A/C, the increase relative to background remained ∼3-fold (Figure 2C). This figure is largely consistent with the increase in frequency of abnormally shaped nuclei observed by PAP staining (Figure 1). We suggest that reduction of BRG1 protein levels in MCF-10A mammary epithelial cells induces a deformation in nuclear shape that can be visualized by any of the three methods utilized.

It is important to note that we observed no evidence for changes in Lamin A/C or Lamin B levels upon BRG1 knockdown (Figure 2A and data not shown), suggesting that the expression of the genes encoding these proteins is not altered by the decrease in BRG1. We also took a candidate approach to examine by immunofluorescence staining the levels and localization of Lamin associated proteins as well as other markers of nuclear structure. No discernable changes in the Lamin associated proteins, Emerin, Nesprin, and Nurim were observed, nor were any changes in the splicing speckle component SRm160, the nuclear matrix associated nuclear mitotic apparatus protein (NUMA), or the chromatin associated protein high molecular weight group protein N2 (data not shown). In addition, no changes in staining against H1 or the modified histones phospho-H3Ser10 and H3triMeK4 were noted (data not shown).

**Figure 3:**
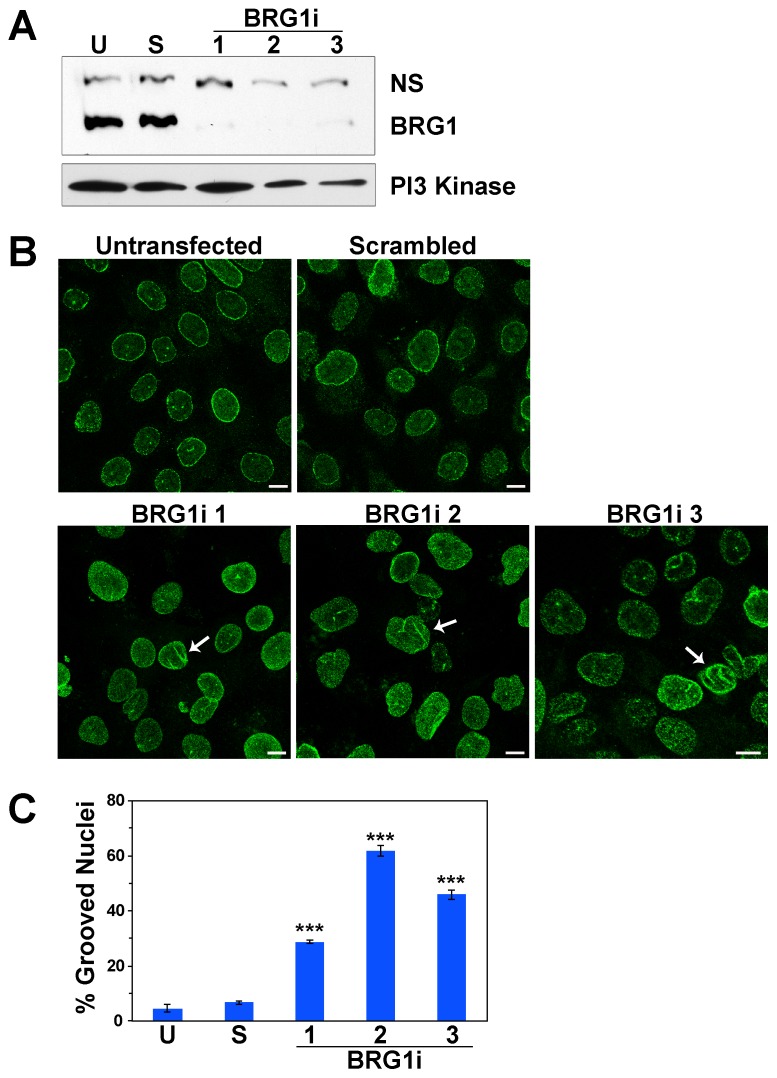
siRNA targeting of BRG1 confirms changes in nuclear shape in MCF-10A cells. (A) Western blot analysis of MCF-10A cells transfected with three different siRNAs that target BRG1 or a control siRNA. U represents untransfected MCF-10A cells. S represents the control cells expressing a scrambled siRNA. 1, 2, and 3 represent the three siRNAs targeting BRG1. PI3 Kinase was measured as a loading control. (B) Untransfected MCF-10A cells, control MCF-10A cells transfected with a scrambled siRNA, and MCF-10A cells transfected with three different siRNAs targeting BRG1 were labeled with Lamin A/C. Arrows indicate grooves. Size bars 10 µm. (C) Quantification of grooved nuclei in untransfected, control, and BRG1 siRNA treated cells. Data represent at least three independent counts of at least 100 nuclei scored for grooves. Results are reported as means +/− standard deviation. *** represents p<0.005 as determined by Student’s t-test whether the BRG1 knockdown results were compared to untransfected or to control siRNA treated cells.

To confirm that the observed effects on nuclear shape were specifically due to a reduction in BRG1 levels, we introduced three different siRNAs targeting BRG1 or a control siRNA into MCF-10A cells. Western blot analysis indicated a greater than 90% reduction in BRG1 protein levels following transfection of each of the BRG1-specific siRNAs (Figure 3A). We then evaluated the transfected cells for changes in nuclear shape. Confocal imaging of Lamin A/C confirmed the presence of nuclear grooves (arrows) (Figure 3B). Quantification indicated that 30% to 60% of the nuclei in cells with siRNA targeting BRG1had grooves, compared to 5% to7% in the untransfected and control scrambled siRNA cells (Figure 3C). This further confirms the observation that a reduction of BRG1 protein levels in MCF-10A cells induces nuclear shape changes.

### Nuclear Structure Is Brg1 Independent In Metastatic Breast Cells

**Figure 4:**
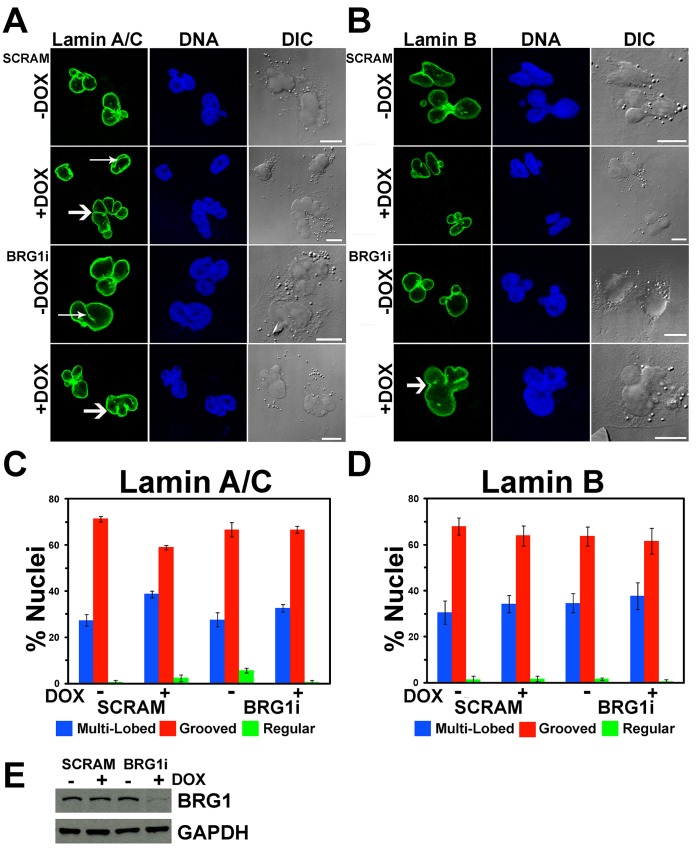
The frequency of grooved and multi-lobed nuclei in MDA-MB-231 cells is independent of BRG1. (A, B) Multiple representative individual confocal sections of MDA-MB-231 cells with and without BRG1 knockdown are shown labeled with either Lamin A/C (A) or Lamin B (B). SCRAM represents the control cells expressing a scrambled sequence shRNA. Nuclei were counterstained with the DNA dye DRAQ5. Arrows indicate nuclear grooves. Thick arrows indicate multi-lobed nuclei. Size bars 10 µm. (C, D) Quantification of regular, grooved, and multi-lobed nuclei indicated no change in MDA-MB-231 cells with or without BRG1 knockdown as determined by staining with Lamin A/C (C) or Lamin B (D). Data represent three counts of at least 100 nuclei scored for grooves under each condition and are reported as the means +/− standard deviation. (E) Western blot analysis of BRG1 levels. GAPDH levels were monitored as a loading control.

The MDA-MB-231 cell line was derived from a metastatic breast adenocarcinoma [38]. Its karyotype is near-triploid but the chromosome content is heterogeneous in the population of cells. MDA-MB-231 cell nuclei are frequently irregularly shaped. MDA-MB-231 cells inducibly expressing shRNA against BRG1 (BRG1i) or a control shRNA (SCRAM) were engineered using the same methodology used to make the MCF-10A cells expressing shRNA targeting BRG1 (manuscript in preparation; see methods). To determine whether BRG1 affected nuclear structure in the context of this metastatic breast cancer cell line, we examined nuclei by Lamin A/C and Lamin B staining (Figure 4A–B). Oval shaped nuclei lacking the lobes, blebs, folds, or grooves previously described were considered “normal”, but such nuclei were observed in, at most, 5% of cells, and in most cases, the percentage of regularly shaped nuclei was much lower (Figure 4C–D). Most nuclei were characterized by prominent grooves, irregular shapes, and/or multiple lobes (Figure 4A–B). Quantification of the number of nuclei containing grooves or multi-lobed nuclei indicated that about one-third contained grooves and approximately two-thirds were multi-lobed. Reduction of BRG1 levels had no effect on the relative proportion of nuclear alterations (Figure 4C–D). Western blot analysis confirmed the inducible knockdown of BRG1 in these cells (Figure 4E). The Catalogue of Somatic Mutations in Cancer (COSMIC) Cell Line Project database indicates that the gene encoding BRG1 is not mutated in MDA-MB-231 cells (http://cancer.sanger.ac.uk/cancergenome/projects/cosmic/). We conclude that reduction of BRG1 in the context of a metastatic breast cancer cell that already shows altered nuclear morphology has no additional effect on nuclear structure.

### Detailed Examination Of Nuclear Structure

**Figure 5:**
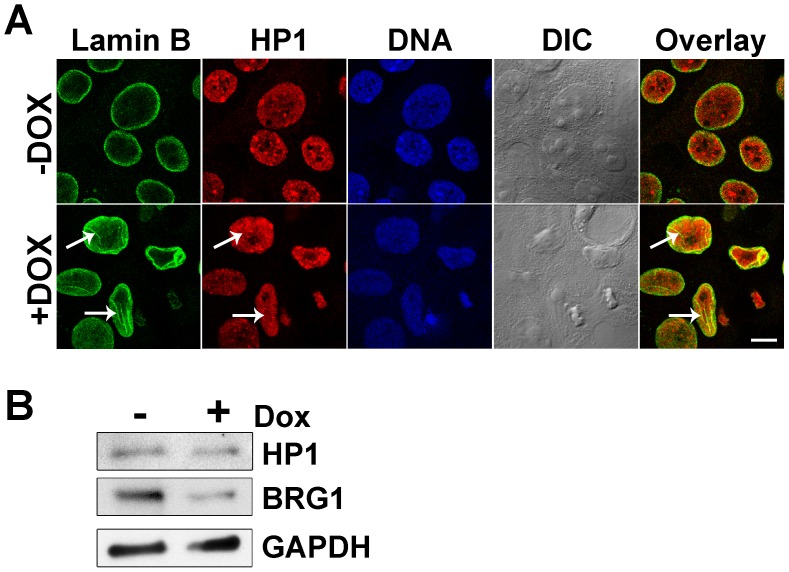
HP1 is not concentrated in nuclear grooves induced by knockdown of BRG1 in MCF-10A cells. (A) Representative and matched individual confocal sections of Lamin B (green) and HP1 (red) stained cells grown in the presence or absence of doxycycline. Nuclei were counterstained with the DNA dye DRAQ5 (blue). Arrows indicate grooves in the Lamin B and Overlay images. Arrows indicate lack of corresponding grooves in the HP1 image. Size bar 10 µm. (B) Western blot analysis of HP1 and BRG1 levels. GAPDH levels were measured as a loading control.

Next, we determined whether there was any spatial relationship between the nuclear grooves and peripheral heterochromatin. Immunofluorescent staining of heterochromatin protein 1 (HP1) indicated that heterochromatin distribution in these cells was not altered by BRG1 knockdown and that there was no preferential localization between HP1 and the nuclear grooves (arrows) (Figure 5A). Despite the lack of any obvious connection between HP1 and BRG1-dependent nuclear grooves, Western blot analysis indicated that cells expressing the BRG1 shRNA contained a slight reduction in HP1 protein levels (Figure 5B).

**Figure 6:**
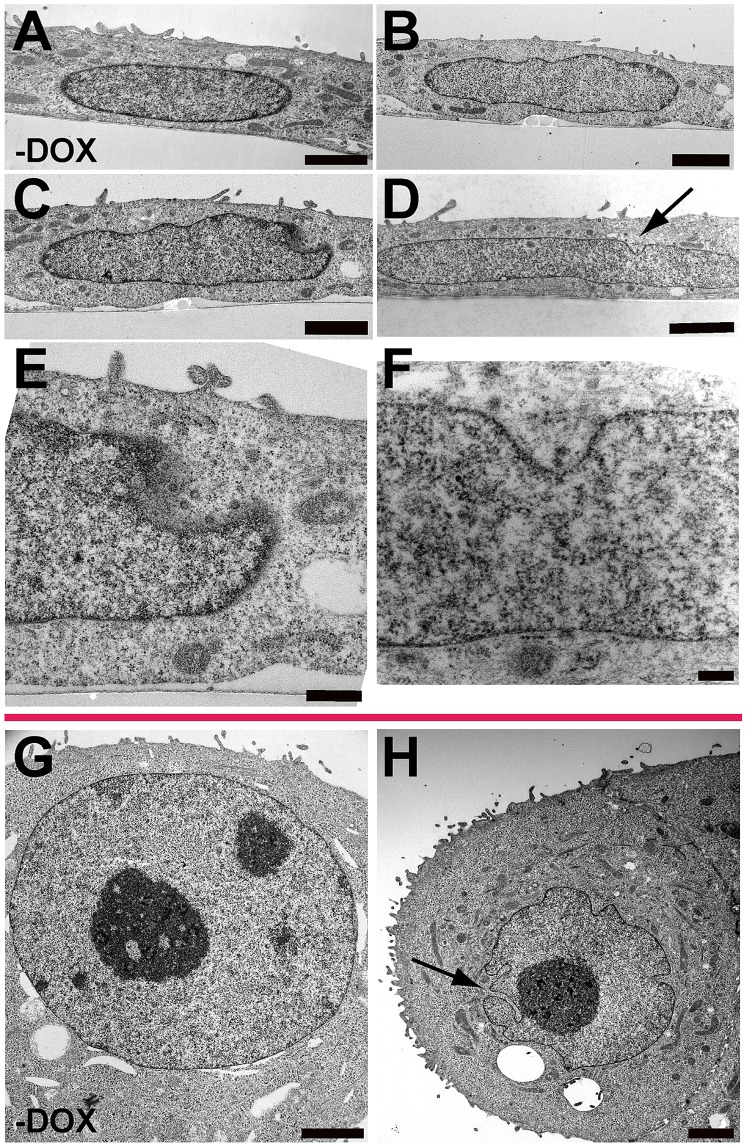
The Ultrastructure of MCF-10A cells with BRG1 knockdown. Sections were prepared for electron microscopy in two orientations: perpendicular to the growth surface (panels A to F) and parallel to the growth surface (panels G and H). Representative images of the structures quantified in Figures 1 and 2 are shown. (A) Uninduced control MCF-10A BRG1i cells had regular oval profiles when viewed perpendicular to the growth plane. For all cells shown in this orientation, the basal side of the cell adjacent to the growth surface is toward the bottom of the micrograph, while the apical surface is facing up. (B) Irregular contours were induced in cells after BRG1 knockdown. (C) After BRG1 knockdown, some nuclei developed both irregular contours and grooves in the apical surface of the nuclei. The groove in this nucleus is shown at higher magnification in panel E. (D) Other nuclei had grooves on their apical sides but a more normal nuclear contour. Arrow indicates groove. The groove for this nucleus is shown at higher magnification in panel F. (E) Higher magnification view of the groove in the nucleus of panel C. Five nuclear pores are visible in the cleft of the groove. This shows that the wall of the groove is in an adjacent plane of section. (F) Higher magnification view of the groove in the nucleus of panel D. (G) Uninduced control MCF-10A BRG1i cells had regular oval profiles when viewed parallel to the growth plane. (H) After induction of BRG1 knockdown, these nuclei sometimes had deep invaginations of the nuclear lamina and covering envelope that projected deep into the nuclear interiors and sometimes terminated adjacent to nucleoli. Arrow indicates groove. Size bar panel E 0.5 µm. All other size bars are 2 µm.

Electron microscopy was used to evaluate the ultrastructure of MCF-10A nuclei after BRG1 knockdown. Sections were prepared in two orientations: parallel to the growth surface and perpendicular to the growth surface. The latter permitted an evaluation of nuclear shape changes at the apical and basal surfaces of the nucleus. This was especially important because the folds in the nuclear lamina that we observed were often on the basal, or top, surface of the nucleus. MCF-10A cells [41] or uninduced MCF-10A BRG1i cells (Figure 6A and G) had regular oval shapes when viewed in either orientation. BRG1 knockdown caused an increase in irregularities in the structures of the nuclear periphery. In sections perpendicular to the growth surface, nuclear shape changes included irregularities in shape and deviations from an oval profile (Figs. 6B and C), and grooves at the top surface of nuclei (Figs. 6C, D, E, and F). Figure 6E presents a higher magnification view of the groove in the nucleus of Figure 6C, while Figure 6F presents a higher magnification view of the groove in the nucleus of Figure 6D (see arrow). In the larger groove of Figure 6E, five nuclear pores are visible, showing that this section is taken near the side wall of the groove, where a more regular profile is resumed. When imaged in sections parallel to the growth surface (Figure 6H), many nuclei had deep lateral invaginations of the nuclear lamina and envelope into the nuclear interior (see arrow). These were deeper than the grooves at the apical surface of the nucleus and sometimes terminated at or near nucleoli. These lateral invaginations were, on average, wider in diameter than the nucleoplasmic reticulum structures reported to connect with nucleoli [46],[47], though we cannot rule out a relationship between the two structures. The invaginations induced by BRG1 knockdown did resemble the nuclear invaginations that we have reported in three dimensional reconstituted basement membrane cultures of MCF-10A cells [41].

### Evaluation Of Nuclear-Cytoplasmic Contacts

The nucleus is connected to the cytoskeleton through direct and indirect contacts with actin filaments, microtubules, and intermediate filaments [18],[21]–[24],[27]–[29]. These contacts keep the nucleus under isometric tension and are expected to be a major determinant of nuclear shape [19],[20],[35]. We hypothesized that altered and unequal tension applied to the nucleus through the cytoskeleton could cause the BRG1 knockdown-induced changes in nuclear profile. Therefore, disruption of the cytoskeleton may suppress nuclear profile changes in BRG1-knockdown cells.

**Figure 7:**
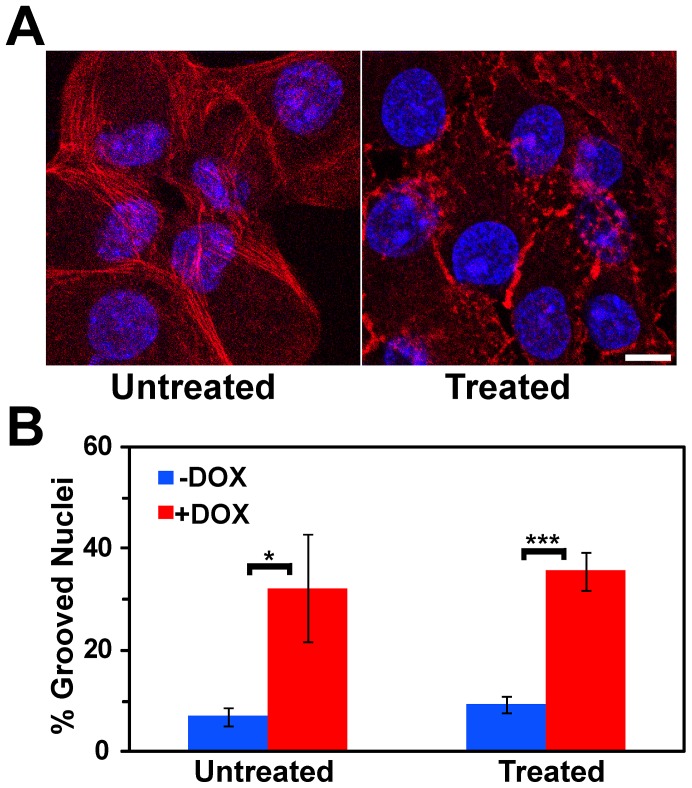
Induction of nuclear grooves after knockdown of BRG1 does not depend on intact actin filaments. (A) Matched individual confocal sections of representative fields of cells before and after treatment with Latrunculin B at 0.2 µg/ml for 30 minutes. Actin filaments were detected with Phalloidin-TRITC (red), while nuclei were counterstained with the DNA dye DRAQ5 (blue). Size bar 10 µm. (B) Quantification of nuclear grooves in control cells and cells treated with Latrunculin B. The means for three counts of at least 100 nuclei scored for grooves under each condition are presented +/− standard deviation. * represents p<0.05, *** represents p<0.005 as determined by Student’s t-test.

Latrunculins are a family of natural products and toxins produced by sponges of the family *latrunculia* [48]. Latrunculin B binds ß-Actin, preventing polymerization and, when added to cells, it depolymerizes actin filaments [49],[50]. It has specifically been shown to disrupt the actin cytoskeleton in MCF10A cells [51]. Figure 7A shows ß-Actin staining in control cells cultured in the absence of doxycycline and in the presence or absence of Latrunculin B. In the untreated cells, long continuous actin fibers were observed throughout the cytoplasm. In treated cells the actin fibers were clearly disrupted (Figure 7A). Latrunculin B treatment did not, however, alter the percentage of cells showing nuclear grooves in either the presence or absence of BRG1 knockdown (Figure 7B).

**Figure 8:**
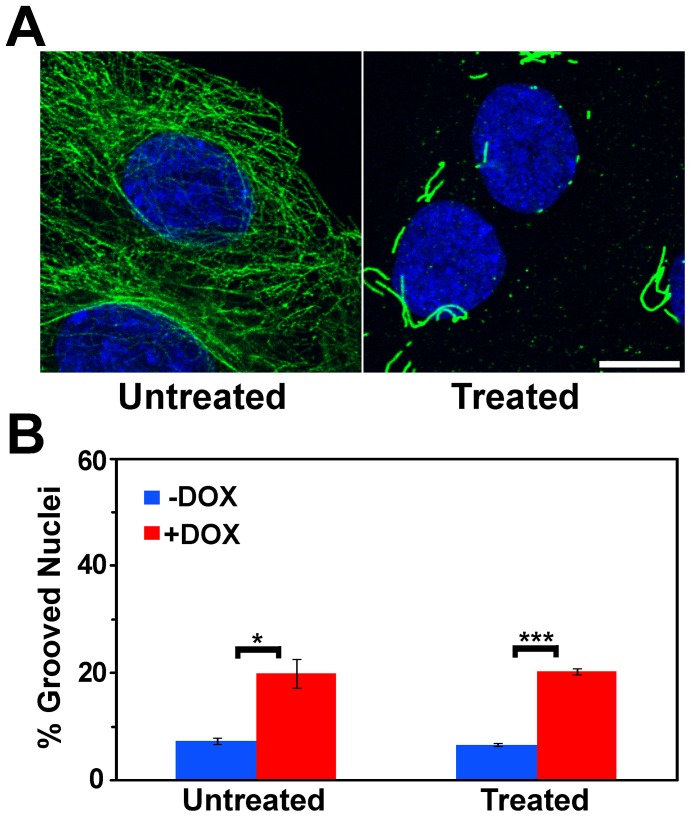
Induction of nuclear grooves after knockdown of BRG1 does not depend on intact microtubules. (A) Matched individual confocal sections of representative fields of cells before and after treatment with Colcemid at 0.05 µg/ml for 3 hours. Microtubules were detected with an α-Tubulin antibody (green), while nuclei were counterstained with the DNA dye DRAQ5 (blue). Size bar 10 µm. (B) Quantification of nuclear grooves in cells treated with Colcemid. The means for three counts of at least 100 nuclei scored for grooves under each condition are presented +/− standard deviation. * represents p<0.05, *** represents p<0.005 as determined by Student’s t-test.

Colcemid depolymerizes microtubules and prevents new microtubule polymerization [52],[53]. Microtubules seen in untreated cells were almost completely disrupted by colcemid treatment (Figure 8A). Colcemid treatment to disrupt microtubules did not change the frequency of grooves upon BRG1 knockdown (Figure 8B).

**Figure 9:**
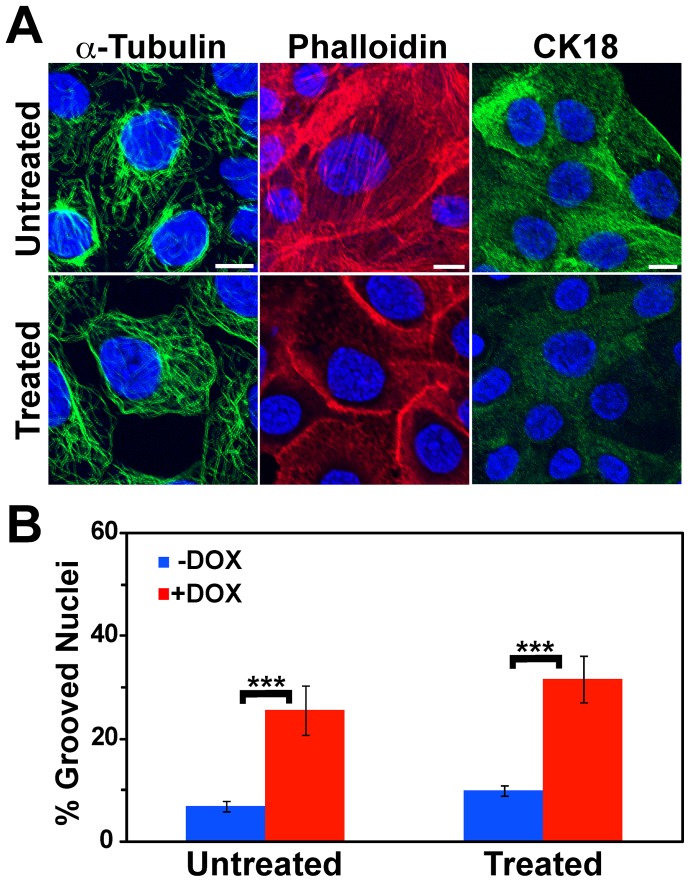
Induction of nuclear grooves after BRG1 knockdown is unaffected by disruption of the cytokeratin network. (A) Matched individual confocal sections of representative fields of cells before and after treatment with orthovanadate at 0.2 µg/ml for 30 minutes. Microtubules, actin filaments, and cytokeratin filaments were detected with an α-Tubulin antibody (left column), Phalloidin-TRITC (center column), and with a Cytokeratin 18 (CK18) antibody (right column), respectively. Nuclei were counterstained with the DNA dye DRAQ5 (blue). Size bars 10 µm. (B) Quantification of nuclear grooves in cells treated with orthovanadate. The means for three counts of at least 100 nuclei scored for grooves under each condition are presented +/− standard deviation. *** represents p<0.005 as determined by Student’s t-test.

The nucleus is also connected to intermediate filaments, which in epithelial cells such as MCF-10A are heteropolymers of cytokeratins. Sodium orthovanadate is a tyrosine phosphatase inhibitor [54] that has been shown to induce transient and reversible alterations in the cytokeratin filament network [55]. Orthovanadate treatment of cells disrupted both intermediate filaments (Figure 9A-α-Tubulin) and actin filament organization (Figure 9A-Phalloidin) and induced a rearrangement of the microtubule network (Figure 9A–CK18). While orthovanadate treatment caused a slight increase in the percentage of cells in the population containing nuclear grooves, the increase occurred both in the presence and absence of BRG1 knockdown. Differences between untreated and treated cells were not statistically significant, and the ratio of cells containing grooves in BRG1 knockdown cells to control cells was unchanged after orthovanadate treatment (Figure 9B). We conclude that disruption of the major cytoskeletal filament systems in mammary epithelial cells does not mediate BRG1-dependent structural alterations in the nuclear periphery.

## Discussion

### The Role Of Brg1 In Regulating Nuclear Shape

BRG1 and BRM are the alternative ATPase subunits for SWI/SNF chromatin remodeling enzymes [56]. Depletion of either BRG1 or BRM causes a slow growth phenotype in non-tumorigenic MCF-10A mammary epithelial cells [8]. We report here that depletion of BRG1, but not of BRM, induced nuclear shape changes in MCF-10A cells. These changes in nuclear shape included folding in the structures at the nuclear periphery, the nuclear lamina and envelope, and lobulation. Similar changes have been observed in patients with laminopathies, diseases caused by the mutation of Lamin A or its interacting structural proteins [15],[16].

The determinants of nuclear shape are poorly understood. The nucleus in differentiated cells is under tension mediated by a pre-stressed cytoskeleton [19],[57]. The balance of forces delivered to the nucleus in this way may account for the great variety of nuclear shapes observed in tissues.

In this report, we tested the hypothesis that changes in nuclear shape that follow BRG1 reduction are mediated by nuclear tension. This hypothesis was consistent with published work on nuclear-cytoskeletal connections and also with an intriguing literature on the connections between BRG1 and the control of cell size and shape. Re-introduction of BRG1 into BRG1 deficient adenocarcinoma cell lines not only induced cell cycle arrest but also caused the cells to assume a flatter and larger appearance with an increased area of surface attachment [58]–[61]. BRG1 deficient adenocarcinoma cells expressing an ATPase defective BRG1 induced a “flat cell” phenotype at a significantly reduced frequency [58],[61], suggesting that the change in cell morphology required BRG1 enzymatic activity. The observed morphology change was attributed to BRG1-dependent organization of actin filaments [60]. However other studies employing inducible expression of the same ATPase-deficient BRG1 allele in NIH3T3-derived mouse fibroblasts reported a similar “flat cell” morphology that involved altered focal adhesions but apparently normal actin organization [62]. Thus there are likely multiple mechanisms by which BRG1 controls cell morphology. These previous results are all consistent with the hypothesis that BRG1-related changes in the cytoskeleton also may cause changes in nuclear morphology. This idea is further supported by the observation that interference with SWI/SNF enzyme function via expression of a dominant negative, ATPase-deficient BRG1 resulted in increased nuclear size [62].

Despite the attractiveness of the working hypothesis, the results were inconsistent, suggesting that internal nuclear mechanisms are responsible for BRG1-mediated shape changes. The concept that epigenetic regulators might help maintain the structural integrity of nuclei is appealing because most of these proteins and complexes have the capacity to alter chromatin structure and because chromatin is mechanically coupled to the nuclear lamina. Chromatin modifying proteins that post-translationally add or remove methyl, acetyl, phospho, ubiquitin, or ADP-ribose groups, among others, have an obvious potential to locally, and perhaps globally, affect chromatin structure and organization [63]. ATP-dependent chromatin remodeling enzymes have the capacity to exert force. Some of this force is used to reorganize chromatin structure by breaking histone:DNA contacts to allow nucleosome movement along DNA, histone displacement, and/or replacement of histones with variant histone counterparts [3]. SWI/SNF enzymes are the prototype ATP-dependent chromatin remodelers in higher eukaryotes; these enzymes have been shown to alter chromatin structure both locally, as demonstrated by changes in chromatin accessibility at target gene regulatory sequences, as well as at a distance, as shown by a dependence on the ATPase subunit BRG1 for intra-chromosomal looping and inter-chromosomal interactions [64]–[67]. BRG1 also contributes to gene expression and may also indirectly affect chromatin and nuclear structure by altering the levels of chromatin components, such as the modest changes in HP1 levels that were observed (Figure 5B). Additional studies will be needed to address the possibility that the induced changes in nuclear structure are due to forces internal to the nucleus caused by changes in chromatin organization.

### Nuclear Shape Changes And Connections To Cancer

Nuclear surface folds and lobulation have been observed in cancer, including breast tumors where alterations in nuclear structure are important diagnostic and prognostic markers [12]–[14]. Despite the dependence of cancer diagnostics on microscopic evaluation of patient biopsies, our understanding of the molecular basis for these cellular structural changes is rather poor. In particular, the molecular mechanisms underlying these changes in nuclear structure remain enigmatic. Although not explained in molecular detail, changes in the shape of nuclei in tumors have been observed since the 1860′s [68]. Irregular nuclear contours in tumor cells often correlate with altered spatial organization of chromatin [12]. Diagnostically useful nuclear features include lobulation, the development of grooves, clefts, or folds, changes in nuclear size, alterations in heterochromatin organization and increases or decreases in the number or appearance of nucleoli. The set of nuclear alterations observed clinically is characteristic of particular tumor types, and not shared by all tumors. The degree of nuclear contour irregularity observed in breast tumors correlates with loss of progesterone receptor expression and with disease progression [13]. Nuclear contour irregularities also predict disease relapse in node negative breast cancer patients [14].

Here we demonstrate that the BRG1 ATPase of an ATP dependent chromatin remodeling enzyme has the capability of regulating nuclear shape. Intriguingly, there are numerous connections between BRG1 and cancer. BRG1 deficient mice are early-embryonic lethal but ∼10% of BRG1 heterozygous mice present with mammary tumors [9],[11]. Primary human breast, lung, pancreas, colon, and prostate tumor samples and/or cell lines derived from such tumors have been reported to contain BRG1 alleles that are deleted, mutated or silenced (reviewed in [69],[70]), further supporting the idea that BRG1 functions as a tumor suppressor. However, other reports indicate that BRG1 expression is elevated in primary tumors and that BRG1 knockdown reduces cell proliferation [71]–[76]. The correlation between inhibition of cell proliferation and BRG1 extends to non-transformed cells as well [8],[77]. Thus the requirement for BRG1 in cellular proliferation and cell transformation is complex and may be context-dependent.

Similarly, the relationship between BRG1 and control of nuclear shape is likely complex and may be context-dependent. BRG1 knockdown in immortalized but non-transformed mammary epithelial cells induces nuclear structural changes reminiscent of changes seen in tumors (Figures 1 and 2) yet was shown to decrease cell proliferation [8]. BRG1 may therefore control the delicate balance between hypo- and hyper-proliferative states. However, there may be limits to the ability of BRG1 to maintain this balance. The MDA-MB-231 cells generated from a metastasized and drug-resistant breast tumor [38] had extremely distorted nuclear profiles, and we did not see significant further alterations after BRG1 reduction (Figure 4). While it may be that BRG1 effects on nuclear shape are lost after malignant transformation, it is also possible that the perturbation of nuclear architecture in MDA-MB-231 cells is already so severe that subtle additional changes could not be distinguished.

## Summary

We have shown that BRG1-dependent changes in nuclear structure do not involve cytoskeletal tension mediated through nuclear-cytoskeletal contacts. We propose that instead, alterations in nuclear structure are generated from within the nucleus as a result of deficiencies in normal chromatin remodeling and organization by BRG1. Our results have implications in understanding the mechanisms involved in nuclear shape changes in diseases such as laminopathies and cancer [12],[15],[16] as well as in development [57],[78].
